# Functional positioning in robotic medial unicompartmental knee arthroplasty: a step-by-step technique

**DOI:** 10.1051/sicotj/2025028

**Published:** 2025-06-11

**Authors:** Luca Andriollo, Giovan Giuseppe Mazzella, Christos Koutserimpas, Pietro Gregori, Elvire Servien, Cécile Batailler, Sébastien Lustig

**Affiliations:** 1 Orthopaedics Surgery and Sports Medicine Department, FIFA Medical Center of Excellence, Croix-Rousse Hospital, Hospices Civils de Lyon, Lyon North University Hospital 69004 Lyon France; 2 Ortopedia e Traumatologia, Fondazione Poliambulanza Istituto Ospedaliero 25124 Brescia Italy; 3 School of Rehabilitation Health Sciences, University of Patras 26504 Rio Greece; 4 Fondazione Policlinico Universitario Campus Bio-Medico 00128 Roma Italy; 5 LIBM-EA 7424, Interuniversity Laboratory of Biology of Mobility, Claude Bernard Lyon 1 University 69100 Lyon France; 6 Univ Lyon, Claude Bernard Lyon 1 University, IFSTTAR, LBMC UMR_T9406 69622 Lyon France

**Keywords:** Functional positioning, Personalized knee arthroplasty, Robotic knee, Unicompartmental knee arthroplasty, UKA

## Abstract

Unicompartmental knee arthroplasty (UKA) compared to total knee arthroplasty, offers several benefits, though it is associated with a higher revision rate, primarily due to suboptimal implant positioning. Recent advances in robotic-assisted techniques have contributed to more personalized and reproducible procedures. Functional Positioning (FP), a three-dimensional alignment concept, introduces a tailored approach based on a surgical technique that is both effective and reproducible. This article presents a step-by-step surgical technique for medial UKA using FP principles in combination with an image-based robotic system. The technique ensures accurate preoperative planning, real-time intraoperative adjustments, and precise component placement. The key steps of this surgical technique include achieving congruent contact points between the femur and tibia under load across the full range of motion, positioning the implant based on the compliance of the medial soft tissues, planning for a targeted laxity that results in an “eagle-wing” appearance, and the use of robotic tools to map cartilage for optimal resurfacing. Future studies will help refine FP strategies and further optimize outcomes in these patients.

## Introduction

Unicompartmental knee arthroplasty (UKA) accounts for approximately 10% of all knee arthroplasties, with a growing trend in utilization due to its advantages over total knee arthroplasty (TKA) [1, 2]. These include less invasiveness, preservation of bone and ligaments, improved functional outcomes, higher patient satisfaction, and faster recovery [3].

Despite these benefits, the long-term survival of UKA remains a limitation, with studies indicating a higher revision rate in comparison to TKA [1]. The technique is less reproducible than TKA, and inaccurate implant positioning is the main cause of early failure and revision [4, 5]. In this context, image-based robotic systems have been demonstrated to have better accuracy in implant positioning and postoperative limb alignment [6, 7].

The impact of robotic surgery has allowed surgeons to move away from the principles of mechanical alignment in UKA, advancing the personalization concepts introduced by Philippe Cartier, whose approach involved an anatomical tibial cut aligned with the native, pre-arthritic medial and posterior tibial plateau slopes [8, 9].

This surgical technique, referred to as functional positioning (FP), describes a personalized approach to medial UKA that aims to respect knee kinematics and ligament compliance through resurfacing using an image-based robotic system.

## Surgical technique

The surgical technique is demonstrated in Video 1. Indications and contraindications have been previously described in detail by Foissey et al. [10]. The patient is positioned supine, with one arm abducted and supported laterally, while the opposite arm rests on the surgical table. A lateral support pad is placed over the mid-thigh, and a distal pad is used to maintain the knee in 90° of flexion during the procedure.

### Step 1: Pre-operative planning

Pre-operative planning involves positioning the prosthetic implant, a fixed-bearing metal-backed unicompartmental arthroplasty (RESTORIS MCK^®^ partial knee implant system, Stryker^®^, Mahwah, USA), using a dedicated navigation system (Orthomap ASM^®^, Stryker^®^, Mahwah, USA). Planning is based on a preoperative CT scan and the generation of a 3D anatomical model.

The implant position is selected at this stage to match patient-specific bone anatomy and restore constitutional coronal alignment. Intraoperative adjustments are expected.

The objective is to restore the hip-knee-ankle (HKA) angle to between 175° and 180°, a posterior tibial slope ranging from 2° to 10°, a difference in Cartier’s angle of ±3°, and a ±2 mm change in the height of the joint line (JL).

During the refinement of the planning, the aim is to obtain centered contact points between the femoral and tibial implants throughout the full ROM and achieve 1–2 mm of laxity in the operated compartment. The goal when determining the laxity curve at various degrees of flexion is to achieve an “eagle-wing” appearance, with up to 2 mm of laxity in full extension and deep flexion, while ensuring a tighter fit between 40° and 80°, avoiding mid-flexion instability, and maintaining good tightness at 90° of flexion. At this stage, a match should be achieved between the ideal load line of the prosthetic component, visible on the screen, and the anatomical load line of the knee, marked during the kinematic evaluation.

### Step 2: Surgical approach and pins placement

A central skin incision is made, followed by an arthrotomy via a medial mid-vastus approach. At this stage, it is essential to perform a cartilage and ligament check of all compartments.

**Figure 1:**
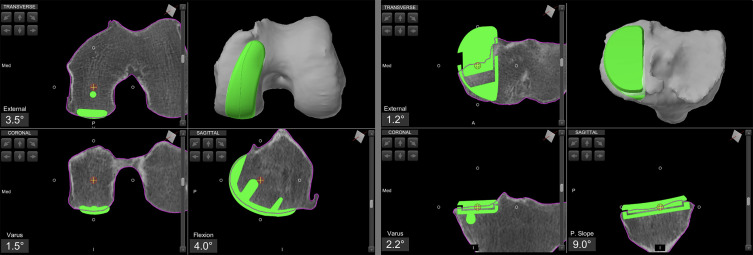
Unicompartmental knee arthroplasty preoperative planning using the Mako^®^ image-based robotic system (Stryker^®^, Mahwah, USA). The case is presented in Video 1.

Robotic assistance is provided using the Mako^®^ System (Stryker^®^, Mahwah, USA). Femoral and tibial pins are placed away from the incision, checkpoints and optical arrays are positioned. The hip center of rotation, bony landmarks, and cartilage thickness are recorded and matched to the pre-operative CT-based model (Figure 1).

### Step 3: Initial knee kinematic evaluation

After bone mapping and matching with the CT imaging, osteophytes from the medial femoral condyle, the margins of the intercondylar notch, and the medial tibial plateau are removed. All overhanging osteophytes should be excised prior to assessing ligamentous compliance, in order to avoid interference with the evaluation of knee kinematics.

After osteophyte removal, kinematic data are acquired, including the range of motion (ROM) and medial soft tissue compliance, through valgus stress. To properly balance the gaps throughout the full ROM and collect femorotibial tracking data, the knee is positioned at different flexion angles, and specific poses are recorded while applying a valgus stress to passively correct the coronal deformity.

### Step 4: Intra-operative planning

During the refinement of the planning, the aim is to obtain centered contact points between the femoral and tibial implants throughout the full ROM and achieve 1–2 mm of laxity in the operated compartment. The goal when determining the laxity curve at various degrees of flexion is to achieve an “eagle-wing” appearance, with 2 mm of laxity in full extension and deep flexion, while ensuring a tighter fit between 40° and 80°, avoiding mid-flexion instability, and maintaining good tightness at 90° of flexion. At this stage, a match should be achieved between the ideal load line of the prosthetic component, visible on the screen, and the anatomical load line of the knee, marked during the kinematic evaluation.

The accuracy of the planning also aims to maintain a neutral sagittal alignment, avoiding fixed flexion or hyperextension.

Determining the correct tibial rotation is challenging, even with the use of CT imaging. This approach involves first assessing the position of the femur by aligning the tibial component with the cortical edge of the femoral condyle in mid-flexion. In medial UKA, the lateral edge of the tibial implant aligns with the axis of the lateral cortex of the medial femoral condyle. Additionally, the alignment of the femoral and tibial implants throughout the entire range of motion is verified intraoperatively and before bone cuts using the 3D planning features of the software.

Final adjustments to the implant placement are made based on the dimensions of the femoral and tibial components (Table 1).

**Table 1:** Functional positioning protocol guidelines in medial unicompartmental knee arthroplasty [HKA: hip-knee-ankle angle; JLO: joint line obliquity; CPAK: coronal plane alignment of the knee].

Parameter	Target
Final coronal alignment (HKA)	175°–180°
Final sagittal alignment with gravity only	0° ± 5°
*Femur*	
Varus/valgus	Femoral resurfacing aims to obtain centered contact points between the femoral and tibial implants throughout the full range of motion
Flexion	
Rotation	
*Tibia*	
Varus/valgus	Cartier’s angle ±3°
Slope	2°–10°
Rotation	Alignment of the implant’s lateral edge with the lateral cortex axis of the medial femoral condyle
JLO and height	JLO orientation to not be changed to different phenotype (CPAK)
	Final joint line height ±2 mm from native
Balancing	1–2 mm of final medial laxity
	Goal: achieve an “eagle-wing” appearance, with 2 mm of laxity in full extension and deep flexion ensure a tighter fit between 40° and 80° avoid mid-flexion instability maintain good tightness at 90° of flexion

**Figure 2:**
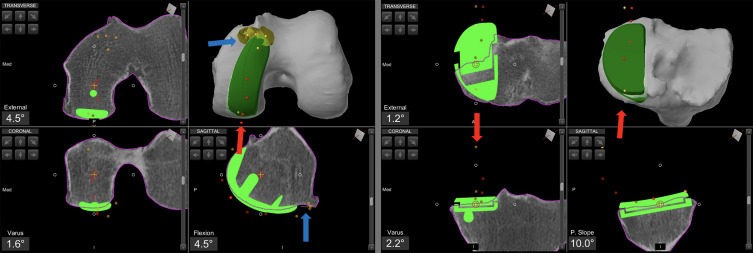
Intraoperative screenshot of medial unicompartmental knee arthroplasty using Mako^®^ (Stryker^®^, Mahwah, USA), with planning modified according to Functional Positioning principles. The blue arrow indicates the points of femoral cartilage mapping, while the red arrow shows the anatomical load line of the knee, marked during the kinematic evaluation.

At the end of the planning, the cartilage is mapped to verify the correct positioning of the components and to avoid overstuffing or understuffing in various planes. A prominent anterior tip of the femoral component can impinge on the patella during flexion, potentially leading to disease progression and pain. Cartilage mapping ensures a smooth transition from the femoral component to the anterior surface of the femoral condyle (Fig. 2).

### Step 5: Bone preparation and trial component implant

**Figure 3:**
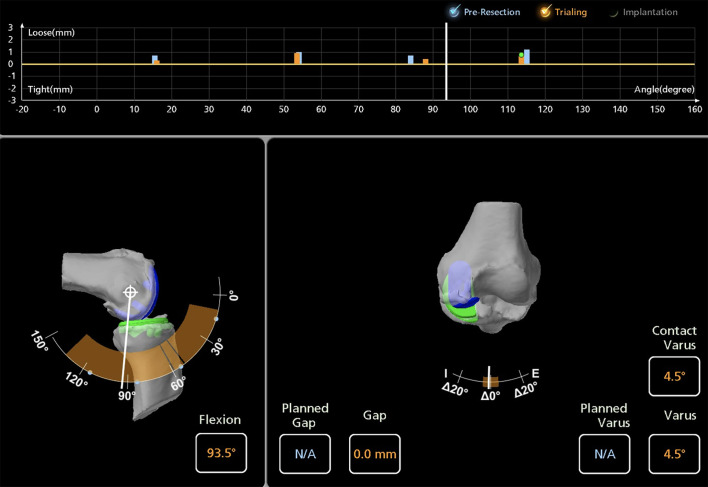
Intraoperative screenshot of medial unicompartmental knee arthroplasty using Mako^®^ (Stryker^®^, Mahwah, USA), with final knee kinematic evaluation.

Bone cuts are performed using the arm-assisted saw and burr. Trial components are inserted, beginning with an 8 mm liner, which may be adjusted to optimize varus-valgus stability. Laxity and knee kinematic alignment are evaluated during the final knee kinematic evaluation, with immediate computer feedback (Fig. 3).

### Step 6: Final implant cementation and closure

The cementation of the final components is performed, with the tibial component cemented first, followed by the femoral component, using progressive compression. Excess cement is removed, and the definitive liner is inserted. After allowing sufficient time for the cement to set, capsular and skin closure are performed.

## Discussion

This article describes and illustrates the application of FP to medial UKA using an image-based robotic system. The role of FP, a three-dimensional alignment concept, is well established in TKA, while this article highlights its emerging application in small implants, particularly in UKA [11]. It is becoming increasingly important to consider not only the patient’s bony anatomy but also joint kinematics and soft tissue compliance [12–14].

Robotic systems have had a significant impact in recent years, with their adoption becoming increasingly widespread in the UKA. This technology enables accurate femorotibial alignment and restores native kinematic alignment, overcoming challenges associated with conventional methods and the biomechanics of the knee compartments [5]. However, while robotic systems provide superior radiological accuracy and improved implant positioning, it is still unclear whether these advancements consistently translate into better functional outcomes [15].

As is well known, key factors in UKA include the correction of varus alignment and the position of the JL [5]. As reported by Foissey et al., a multivariate analysis of 366 patients identified a post-operative HKA ≤ 175° and a JL lowering ≥ 2 mm as significant risk factors for tibial implant failure [5]. Particular attention should be paid to patients with a pre-operative HKA < 172°, as a post-operative HKA < 175° was commonly observed in these knees.

Emerging research supports the idea that advances in personalization within knee arthroplasty may lead to better functional outcomes, greater patient satisfaction, and more natural knee kinematics. When it comes to applying these principles to UKA, however, the long-term durability of such approaches is still under investigation, highlighting the need for further high-quality research.

## Conclusions

This personalized approach, applicable to UKA and referred to as FP, represents a promising advancement in the management of medial osteoarthritis. The key steps of this surgical technique include achieving matching contact points between the femur and tibia under load across different degrees of ROM, positioning based on the compliance of the medial soft tissues, planning for a targeted laxity that produces an “eagle-wing” appearance, and the possibility, through robotic tools, to map cartilage for optimal resurfacing. This surgical technique is both effective and reproducible. Future studies will help refine FP strategies and further optimize outcomes in these patients.

## Data Availability

The video is available at the following URL: https://www.sicot-j.org/10.1051/sicotj/2025028/olm.

## Supplementary material

*Video 1*: Step-by-step surgical technique video of functional positioning in robotic medial unicompartmental knee arthroplasty. The supplementary material is available at https://www.sicot-j.org/10.1051/sicotj/2025028/olm.

## References

[1] Achakri H, Ben-Shlomo Y, Blom A, et al. (2022) The National Joint Registry 20th Annual Report 2023. London: National Joint Registry. 38422195

[2] Sangaletti R, Meschini C, Capece G, et al. (2024) A morphometric medial compartment-specific unicompartmental knee system: 5 years follow up results from a pilot center. Knee 47, 179–185. 38401342 10.1016/j.knee.2024.02.005

[3] Foissey C, Batailler C, Fontalis A, et al. (2024) Long-term outcomes in unicompartmental knee arthroplasty: Survivorship of medial versus lateral unicompartmental knee arthroplasty. J ISAKOS 9, 100329. 39413926 10.1016/j.jisako.2024.100329

[4] Andriollo L, Benazzo F, Cinelli V, et al. (2024) The use of an imageless robotic system in revision of unicompartmental knee arthroplasty. Knee Surg Sports Traumatol Arthrosc 33(5), 1792–1803. 39740128 10.1002/ksa.12574 PMC12022834

[5] Foissey C, Batailler C, Vahabi A, et al. (2023) Combination of a high residual varus and joint-line lowering strongly increases the risk of early implant failure in medial unicompartmental knee arthroplasty. J Arthroplasty 38, 2275–2281. 37271228 10.1016/j.arth.2023.05.055

[6] Gaggiotti S, Foissey C, Pineda T, et al. (2024) Enhancing robotic precision in medial UKA: Image-based robot-assisted system had higher accuracy in implant positioning than imageless robot-assisted system across 292 knees. Knee Surg Sports Traumatol Arthrosc 32, 2097–2106. 38690988 10.1002/ksa.12215

[7] Andriollo L, Montagna A, Mazzella GG, et al. (2024) Navigated versus conventional medial unicompartmental knee arthroplasty: Minimum 18 years clinical outcomes and survivorship of the original Cartier design. Knee 49, 183–191. 39043013 10.1016/j.knee.2024.07.009

[8] Cartier P, Sanouiller J-L, Grelsamer RP (1996) Unicompartmental knee arthroplasty surgery: 10-year minimum follow-up period. J Arthroplasty 11, 782–788. 8934317 10.1016/s0883-5403(96)80177-x

[9] Rivière C, Logishetty K, Villet L, Maillot C (2021) Calipered kinematic alignment technique for implanting a Medial Oxford^®^: A technical note. Orthop Traumatol Surg Res 107, 102859. 33601029 10.1016/j.otsr.2021.102859

[10] Batailler C, Libert T, Oussedik S, et al. (2024) Patello-femoral arthroplasty- indications and contraindications. J ISAKOS 9, 822–828. 38185247 10.1016/j.jisako.2024.01.003

[11] Koutserimpas C, Andriollo L, Gregori P, et al. (2025) Revisiting terminology: The transition from “functional alignment” to “functional knee positioning”. Knee Surg Sports Traumatol Arthrosc. 10.1002/ksa.12667. 40167115

[12] Andriollo L, Koutserimpas C, Gregori P, et al. (2025) A new parameter in the era of robotic total knee arthroplasty: Coronal alignment at 90 of flexion impacts clinical outcomes. Knee Surg Sports Traumatol Arthrosc. 10.1002/ksa.12648. PMC12205417 40099499

[13] Andriollo L, Koutserimpas C, Gregori P, et al. (2025) Beyond the coronal plane in robotic total knee arthroplasty-Part 1: Variations in tibial slope and distal femoral flexion do not affect outcomes. Knee Surg Sports Traumatol Arthrosc. 10.1002/ksa.12658. PMC12310085 40130477

[14] Andriollo L, Gregori P, Koutserimpas C, et al. (2025) Beyond the coronal plane in robotic total knee arthroplasty-Part 2: Combined flexion does not affect outcomes. Knee Surg Sports Traumatol Arthrosc. 10.1002/ksa.12660. PMC12310087 40145260

[15] Jiao X, Du M, Li Q, et al. (2024) Does patient-specific instrument or robot improve imaging and functional outcomes in unicompartmental knee arthroplasty? A Bayesian analysis. Arch Orthop Trauma Surg 144, 4827–4838. 39294530 10.1007/s00402-024-05569-y

